# The promise of biomarkers: precision medicine will pave a roadmap for pediatric acute kidney injury management in critically ill children

**DOI:** 10.1007/s44253-025-00086-1

**Published:** 2025-09-24

**Authors:** Natalja L. Stanski, Jun Oh, Rajit K. Basu

**Affiliations:** 1 Department of Pediatrics, University of Cincinnati College of Medicine, Cincinnati, OH USA; 2 Division of Critical Care Medicine, Cincinnati Children’s Hospital Medical Center, Cincinnati, OH USA; 3 Division of Pediatric Nephrology, University Medical Center Hamburg/Eppendorf, Hamburg, Germany; 4 Division of Critical Care Medicine, The Ann and Robert Lurie Children’s Hospital Medical Center, Chicago, IL USA

**Keywords:** Acute kidney injury, Precision medicine, Biomarkers, Theragnosis, Pediatrics

## Abstract

Acute kidney injury (AKI) is common in critically ill children and neonates and imparts an increased risk for morbidity and mortality. Despite a growing recognition of the untoward consequences of AKI, its management continues to rely on supportive care alone, after numerous clinical trials have failed to identify effective disease-modifying therapies. This failure to advance the field is likely due in large part to the heterogeneity of AKI, which demands a precision approach to diagnosis and management. Despite the emergence of several novel AKI biomarkers with the ability to refine the AKI diagnosis beyond what is afforded by changes in serum creatinine and/or urine output alone, widespread translation of these biomarkers to practice has been limited. In this review, we outline a roadmap for AKI risk-stratification, diagnosis, management, and follow-up that is rooted in precision medicine principles and feasible with the tools currently available in pediatric ICUs. This roadmap highlights the importance of dynamic (as opposed to static) assessment of the critically ill child with, at-risk for, and recovering from AKI, and introduces the concept of theragnostic biomarkers that are both the target of and change with treatment, thus helping guide the therapeutic approach. Finally, we highlight the need for re-defining appropriate endpoints in AKI clinical trials testing the interventions proposed here (and others) to ensure we are identifying treatments that will meaningfully improve outcomes for critically ill children with AKI.

## Introduction

Acute kidney injury (AKI) impacts nearly 1 in 3 critically ill children and neonates during intensive care unit (ICU) admission, imparting increased risk for morbidity and mortality to affected patients, and resulting in increased health care resource utilization and expenditures [1–3]. AKI is now recognized to be a heterogenous syndrome with multiple different subphenotypes (subset of *clinical* features with a shared phenotype [i.e., AKI] that distinguishes the group from others within that phenotype) and endotypes (a subset of patients within a phenotype [i.e., AKI] that share a distinct *biological mechanism* of disease), each of which likely require individualized approaches to management [4–6]. Unfortunately, the diagnosis of AKI currently relies solely on changes in serum creatinine (SCr) or urine output (UOP), both of which afford little granularity or insight into this heterogeneity at the individual patient level [7]. The result of this imprecise diagnostic framework is an equally imprecise (and ineffective) suite of disease-modifying therapies for AKI, forcing clinicians to rely solely upon supportive care for affected patients. Over the past several years, the emergence of novel tubular injury biomarkers has served to resolve some of the heterogeneity of AKI, and affords the possibility of more precise, targeted approaches to management and risk stratification. However, while the 23rd Acute Disease Quality Initiative (ADQI) recommended combining kidney injury biomarkers with functional kidney biomarkers like SCr and UOP to improve diagnostic accuracy for AKI [8], such an approach has not yet been widely implemented in practice. Given the continued poor outcomes for critically ill children with AKI [1, 2, 9], there is a desperate need for more precise risk stratification, diagnosis and management strategies, both to inform clinical care and to guide appropriate patient enrollment in future studies of potential disease-modifying treatments. Toward this end, we provide here a proposed ideal roadmap for AKI care, from risk stratification through follow-up, that employs precision medicine principles and leverages existing and on-the-horizon biomarkers. We will also explore how we bridge the gap between where we are and where we need to be, highlighting the key gaps and priorities moving forward.

## The Ideal Roadmap: From Risk Stratification through Follow-Up

**Fig. 1:**
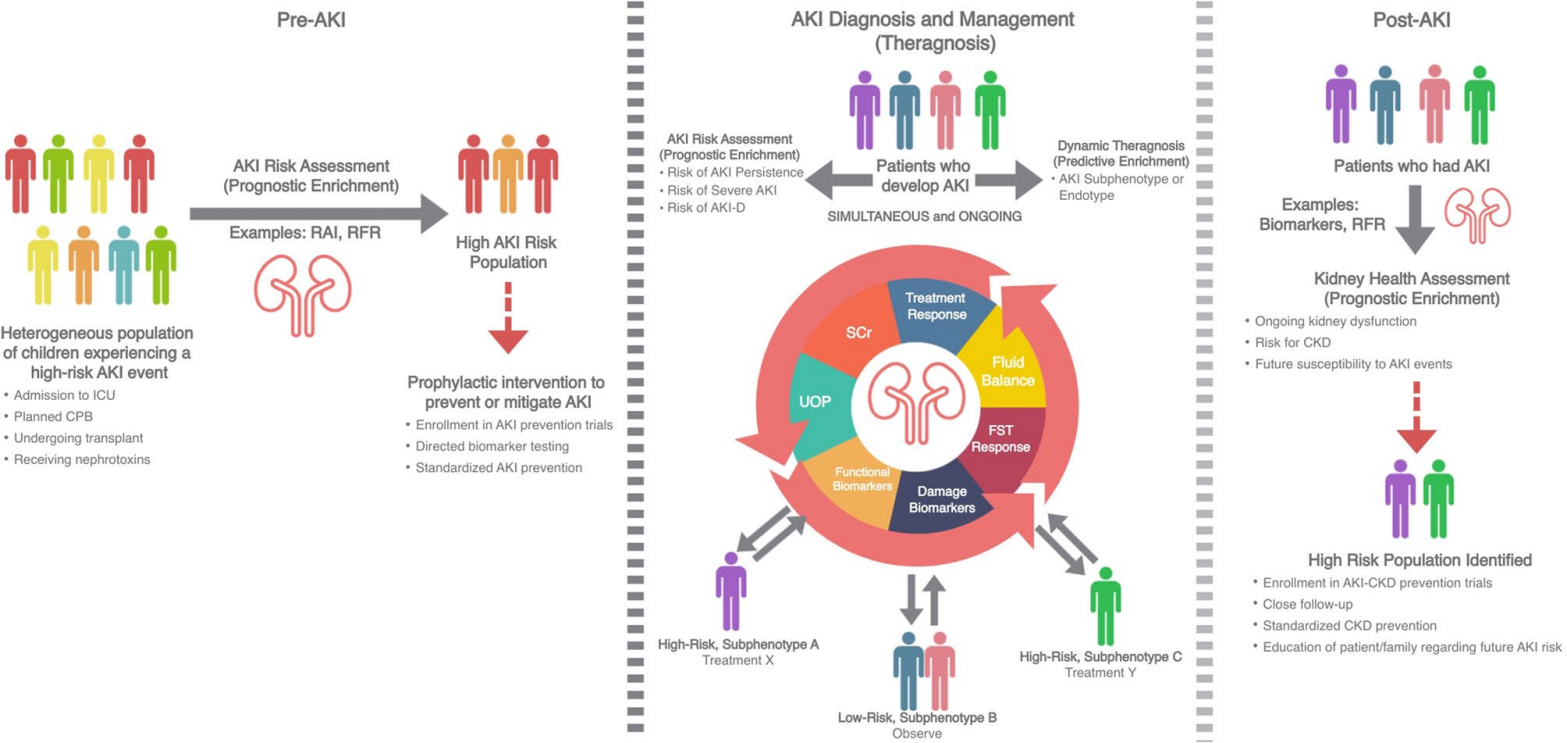
A Proposed Road Map for Precision Pediatric Acute Kidney Injury (AKI) Care in Critically Ill Children. Abbreviations: CPB- cardiopulmonary bypass; RAI- renal angina index; RFR- renal functional reserve; AKI-D- AKI requiring dialysis/renal replacement therapy; FST- furosemide stress test. SCr- serum creatinine; UOP- urine output; CKD- chronic kidney disease

The ideal management approach to heterogeneous syndromes like AKI is based in precision medicine principles. Broadly, precision medicine refers to prevention diagnostic, and treatment strategies that take individual patient characteristics into account [6, 10]. Within this framework lies the concept of enrichment, which generally refers to the selection of a subgroup of patients more likely to have a disease-related outcome of interest (i.e., prognostic enrichment) or more likely to respond to a given therapy (i.e., predictive enrichment) [6, 10]. Thus, the ideal roadmap for AKI management will leverage existing tools and biomarkers to both (1) risk-stratify patients for relevant outcomes of interest (i.e., AKI development or persistence, progression to CKD) to guide further testing or intervention (i.e., prognostic enrichment), and (2) to better characterize the AKI subphenotype/endotype to guide specific therapies (i.e., predictive enrichment). This ideal roadmap, which serves as the foundation for the subsequent sections below, is outlined in Fig. 1. For purposes of this review, we will utilize the term subphenotype and endotype interchangeably for simplicity, as a particular patient may have overlapping subphenotype(s) and endotype(s) concurrently [6].

## Pediatric AKI risk stratification: a window of opportunity?

The 26th ADQI focused on pediatric AKI recommends comprehensive AKI risk assessment in children at time of or prior to hospital admission, with the latter occurring when future admissions are scheduled (i.e., surgery) [4, 5]. In pediatrics, the best evidence in support of this practice has been demonstrated in the ICU, where the Trial in AKI using NGAL and Fluid Overload to optimize CRRT use (TAKING FOCUS 2; TF2) demonstrated that a sequential risk stratification process including the Renal Angina Index (RAI) calculated 12 h after pediatric ICU admission followed by urine neutrophil gelatinase-associated lipocalin (uNGAL) measurement in patients identified as high risk both accurately predicted severe AKI and improved patient outcomes [11, 12]. While no care was mandated as part of the TF2 algorithm, the process of multi-modal risk stratification (i.e., prognostic enrichment) was associated with clinical decision support to personalize fluid management practices, including initiation of continuous renal replacement therapy (CRRT) when appropriate [11, 12]. Similarly, in critically ill adult populations following surgery, biomarker-based risk stratification using the product of urinary tissue inhibitor of metalloproteinases-2 and insulin-like growth factor binding protein 7 ([TIMP2]●[IGFBP7]) to guide the implementation of standardized AKI care bundles reduced the frequency and severity of AKI compared to standard care [13, 14]. While these two examples employ well-validated risk-stratification tools (i.e., RAI) and biomarkers (i.e., uNGAL and [TIMP2]●[IGFBP7]) in their respective populations, additional population-specific examples of emerging, yet-to-be widely validated risk stratification tools for pediatric AKI also exist [15–23]. Finally, though data have yet to be published in children, there is a renewed interest in the use of renal functional reserve (RFR) as a measure of kidney fitness ahead of a planned insult (i.e., cardiopulmonary bypass) [24]. RFR measures the increase in glomerular filtration rate (GFR) in response to a protein load, with poor response indicating reduced RFR and possible subclinical kidney disease [24]. Studies in adult patients undergoing elective cardiac surgery have demonstrated that (1) reduced pre-operative RFR is predictive of AKI in the first week following cardiac surgery [25, 26], and (2) many patients who developed post-operative AKI but recovered without evidence of CKD had reduced RFR at 3 months [27]. These complementary findings highlight the importance of using novel risk stratification tools to identify at-risk populations who might otherwise be missed using standard clinical tools. A comprehensive outline of the available and on-the-horizon pediatric AKI risk stratification tools for critically ill children are listed in Table 1.

**Table 1:** Selection of available and on-the-horizon pediatric acute kidney injury risk stratification tools for critically ill children

Tool	Timing	Endpoint Predicted	Pediatric Evidence/Gaps
General Populations of Critically Ill Children			
Renal Angina Index	12 h after PICU admission	Severe AKI at Day 3	Recent metanalysis demonstrated a pooled AUROC of 0.88 (0.85–0.91) [28]. Validated in multiple cohorts of pediatric critically ill patients (including the specific populations below).
Fluid Overload and Kidney Injury Score (FOKIS)	Continuously calculated during ICU admission	PICU length of stay, mortality	Increasing FOKIS was independently associated with length of stay and mortality after accounting for potential confounders. [23] Has not been externally in other cohorts. Unique because it associates poor outcomes to degree of kidney injury.
Urine Neutrophil Gelatinase-Associated Lipocalin (NGAL)	First 24 h of ICU admission	Severe AKI 48–72 h after admission	In high-risk patients, a cut-point of 125 ng/ml in the first 24 h after ICU admission is predictive of the primary endpoint (AUROC 0.83, 95%CI 0.77–0.90) with high specificity (6%) and NPV (97%) [29]. Multiple studies in children demonstrating its predictive performance.
Sepsis			
Sepsis Renal Angina Index	12–24 h after septic shock	Severe AKI at Day 3 of septic shock	AUROC 0.9 (0.86–0.93) for prediction of primary outcome; specificity improves by using higher threshold (≥ 20) and adding platelet count [18]; has been validated in another multicenter cohort [17].
PERSEVERE-II AKI Prediction Model	12–24 h after septic shock	Severe AKI at Day 3 of septic shock	AUROC 0.95 (0.92–0.98) for primary outcome [15]; has been validated with similar test characteristics in another multicenter cohort [16].
Cardiopulmonary Bypass			
Cardiac Renal Angina Index	8 h post-operatively	Day 3 severe AKI, mechanical ventilation > 7 days, CICU length of stay > 14 days, or mortality.	Multi-center study derived this tool with an AUROC 0.7 (0.62–0.78) for the primary endpoint [19]. Has not yet been externally validated.
Renal Functional Reserve	Pre-operative measurement	AKI in the first 7 days after surgery	In adults, reduced pre-operative RFR is associated with more post-operative AKI [25, 26]. No evidence yet in children.
Fibroblast growth factor 23	Pre- and post-operatively (4–24 h after)	Severe AKI in the first 7 days following surgery	Pre: higher levels in those who developed severe AKI following surgery; no difference in pre-operative SCr, cystatin C or other urinary biomarkers [21, 30] Post: AUROC 0.74 (0.5–0.9) for severe AKI measured 4–8 h after [31]; higher at 12–24 h in those who developed severe AKI [21]. No multi-center studies have been performed in children.
Uromodulin	Pre- and post-operative (24 h after)	AKI at 48 h [31] or 7 days following surgery [22]	Pre: Patients in the lowest quartile of pre-operative urine uromodulin had 132x increased risk of postoperative AKI [22]. Post: 24-hour urine uromodulin predicted 48 h AKI AUROC 0.90 (0.82–0.98) and high specificity (91%) [32] No multi-center studies have been performed in children.
Pediatric Emergency Department (ED)			
Acute Renal Angina Index	Calculated from variables during ED course	aRAI score ≥ 8 to predict any KDIGO AKI (SCr > 1.5x baseline) 24–72 h after admission	One single-center study with AUROC 0.92 (0.86–0.98); outperformed any biomarker (including uNGAL) [20]. Has not yet been externally validated.

Synthesizing the evidence, we propose that a precision-medicine informed ideal roadmap for AKI management in critically ill children would start with patient-, pathology- and context-specific risk stratification (Fig. 1). This risk stratification would occur ahead of a planned insult necessitating critical care (i.e., planned cardiac surgery, bone marrow transplant), at time of admission, with clinical status changes (i.e., patient is intubated, has a new vasoactive medication requirement, or is prescribed a new nephrotoxic medication), and should continue on a rolling basis given the dynamic nature of pediatric critical illness. Importantly, a comprehensive AKI risk stratification process should also continue through discharge, to facilitate appropriate, targeted post-AKI care and follow up. This ideal, personalized, and dynamic AKI risk stratification schema is illustrated in Fig. 1.

## AKI diagnosis and management: leveraging biomarkers to move to theragnosis

Though the Kidney Disease Improving Global Outcomes (KDIGO) guidelines provide a universally accessible and easy to follow foundation for AKI diagnosis, they are hampered by the well-known limitations of functional markers like SCr and UOP, particularly in critically ill children [33]. Biomarkers of kidney damage can identify ongoing injury in different segments of the nephron and may also represent different mechanisms of injury but have been hampered by a monocular approach to implementation and utilization [34]. Moving forward, utilizing functional markers like SCr and UOP with the added narrative of dynamic changes in other biomarkers will likely facilitate the transition from mere diagnosis to guidance for management (i.e., theragnosis) [6, 35]. Below, we outline examples of existing biomarkers and constructs that could be leveraged into a more personalized approach to theragnosis for AKI.

### Creatinine current and beyond

Available evidence has handcuffed the progress of AKI diagnostics to improve care. Use of only linear measurements to assess change in SCr from baseline or between two points has specific limitations in children – given the variability in steady state, muscle mass, sex, and delays in rise from injury. UOP changes are also predicated on a standard of a “baseline” (i.e., 1 ml/kg/hr) which does not incorporate conservation of mass and balance of fluid states. Therefore, both changes in SCr and perturbations in UOP, though indicative of “something wrong”, do very little to elucidate the underlying biological processes of kidney injury [5]. As mentioned earlier, a step forward for utilization of SCr change and the KDIGO recommendations would be tailored use in high-risk patients. Additionally, analysis of SCr changes by rate of change, not just quantity of change, as demonstrated in the kinetic eGFR data [36, 37], would offer more perspective on how the patient and the SCr are moving through illness concurrently. In this way, SCr becomes a “theragnostic” – a marker which is a target of, and changes in relation to, therapy [38]. Additionally, as a solute, SCr is significantly influenced by volume of distribution and should be adjudicated by fluid balance. As demonstrated initially by Macedo and reproduced in numerous adult and pediatric data sets [39–44], correcting SCr for fluid balance is pragmatic, logical, matches physiology, and indirectly integrates changes in UOP (i.e., reduced urine output as a sign of AKI is manifest commonly as higher fluid accumulation which then dilutes SCr). Refining the change in SCr for the confounding variable of fluid balance is therefore a physiologically justified practice with relevance in critical care. Pediatrics has led the way towards appreciation of fluid balance during critical illness – set initially against the backdrop of the timing of RRT initiation [45]. The phases of critical illness and fluid balance – the “ROSE” paradigm (Resuscitation, Optimization, Stabilization, Evacuation) – have now moved the focus of recognizing changes in fluid balance towards distinct windows of time during the patient’s illness course [46]. In addition to adjudicating SCr for fluid balance, using fluid balance as an additional vital sign (akin to heart rate, patient weight, oxygen saturation) and marker of kidney function (along with SCr and UOP) is a dynamic measure to facilitate therapeutic decision making. Further, integration of fluid balance theragnostic measures – such as the urine flow rate over time after a dose of diuretic with a “furosemide stress test” – is a practical and pragmatic utilization of fluid balance to adjudicate kidney function and tubular health [47–49].

### Injury and functional biomarkers for diagnosis and theragnosis

**Fig. 2:**
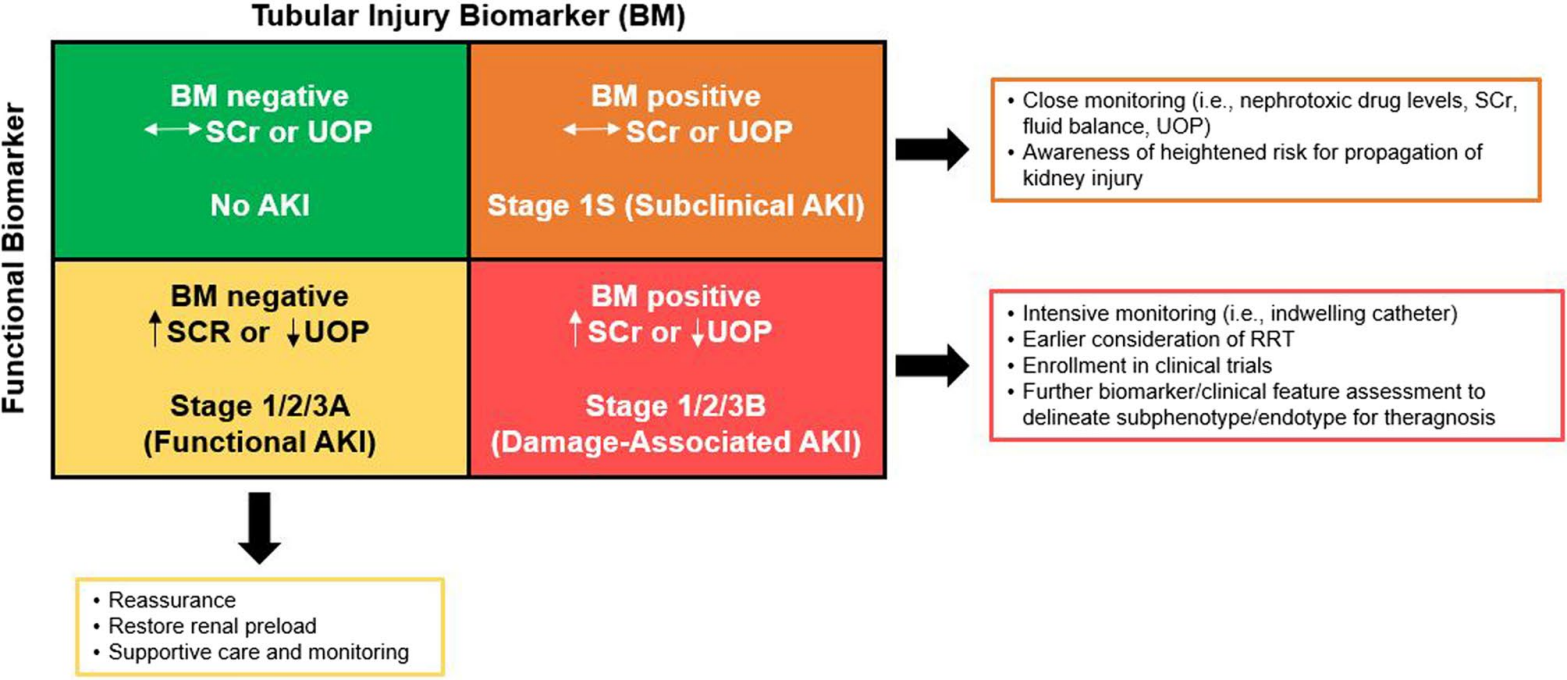
Integration of Functional and Tubular Injury Biomarkers to Refine Acute Kidney Injury (AKI) Staging and Facilitate Theragnosis

Although a wide array of AKI biomarkers, derived initially in animal models and validated in isolated Human populations, have demonstrated high sensitivity and specificity for AKI, integration into practice has been stalled. Over 25 years of research has led to compendiums of data which repeatedly offer statistical evidence of ‘predictive’ efficacy of these biomarkers for severe AKI, particularly when compared to changes in SCr [8, 35]. Additionally, distinct from SCr, tubular injury biomarkers may localize to both location within the kidney and injury mechanisms – offering an unprecedented level of injury endotyping [35, 50]. Despite the robust signals, consistent demonstration of the value-add related to integrating biomarkers into clinical care has not been reported in a widespread fashion to bolster uptake and use. The fundamental question which has evaded answer has been “how does management change if [these] biomarkers are used in clinical care”? The answers could be quite simple to complex. To start, pathophysiological changes in the kidney which occur in acute illness can be detected by some of the biomarkers – detectable hours or even days before SCr levels rise are detected or UOP criteria are met [5, 51–54]. Of note, the changes can be delineated into functional change (previously denoted as related to perfusion of the glomerulus and filtration function before the proximal tubule) and/or tubular damage (related to tubular handling of solute and fluid) [34, 35]. Though not the focus of this review, examples of exisitng functional and tubular injury biomarkers with evidence and clinical availability in critically ill pediatric populations are outlined in Table 2, while a schematic of their proposed integration (described below) is illustrated in Fig. 2. A simple signal which has garnered more attention in the past five years has been the documentation of “subclinical” AKI – denoted by changes in tubular damage biomarkers ahead of or without the change in functional markers (SCr included) [5, 51–55]. Conversely, the identification of “functional” AKI – changes in functional markers (SCr included) without evidence of tubular damage (negative or low tubular biomarker values) – has separated patients with AKI previously denoted as “pre-renal” AKI. Finally, the combination of both functional and tubular damage markers together has created a fourth distinct clinical patient population – previously denoted as patients with “intrinsic AKI” or “acute tubular necrosis”. Integrating these markers to enhance diagnosis is crucial and was highlighted by the ADQI 23 consensus meeting related to refining the diagnostic criteria to include biomarker detected injury [8].

**Table 2:** Selection of available and on-the-horizon pediatric acute kidney injury functional, stress and tubular injury biomarkers

AKI Biomarker	Biological Role	Pediatric Literature	Limitations
Functional Biomarkers			
Plasma Proenkephalin A (PENK)	Endogenous hormone that is freely filtered at the glomerulus.	One study on 97 critically ill children and neonates demonstrated PENK levels highly correlated with iohexol-based measured GFR, and better correlated than cystatin C or SCr [56].	Limited pediatric data Not clinically available
Plasma Cystatin C	Inhibitor of cysteine proteases that is freely filtered at the glomerulus.	Most literature in the pediatric cardiac surgery population. Has been shown in some studies to be an early predictor of AKI [57]. As above, correlation with measured GFR was only moderate in children, and poor in neonates [56]	Several confounders (age, sex, inflammatory state, CKD etc.) [35]. Poor standardization, variability in availability and turn-around time, high cost.
Stress Biomarkers			
Urine [TIMP2]●[IGFBP7]	Released by tubular epithelial cells to induce cell cycle arrest under times of stress	Limited to the post-CPB [58–61] and post-liver transplant [62] populations. Variable performance for prediction of subsequent severe AKI.	Limited pediatric data Clinical assay (NephroCheck^®^) only approved for ≥ 21 years old.
Urine Dickkopf-3 (DKK3)	Glycoprotein secretion into urine during times of tubular stress	One study of 420 pediatric patients should strong correlation between urine DKK3 and risk for AKI in critically ill children, especially sepsis-associated AKI [63]	Limited pediatric data Not clinically available
Damage Biomarkers			
Urine neutrophil gelatinase-associated lipocalin (NGAL)	Glycoprotein produce by neutrophils, epithelial tissues, and renal tubular cells (at least 3 different types)	Several pediatric studies demonstrating good performance for prediction of severe AKI in hospitalized children [29]; now FDA approved for this purpose. May also provide information regarding AKI subphenotype (i.e., damage-associated AKI) that may be helpful for prognosis and to guide therapy [54, 64–66].	Can be elevated in sepsis and other inflammatory states, UTI, CKD; clinically relevant cut-off for AKI may be different in these populations. More data needed on serial use for theragnosis.
Urine C-C motif chemokine ligand 14 (CCL14)	Inflammatory chemokine involved in tissue injury and repair.	Only one small study in pediatric patients following cardiac surgery, no association between CCL14 and persistent severe AKI [67].	Limited pediatric data Clinical assay (NephroCLeaR™) CE-marked for adults in Europe. No FDA approval for adults or children in US.
Urine olfactomedin-4 (OLFM4)	Secreted glycoprotein expressed on mature neutrophils and some epithelial cells following stress. Localizes specifically to the loop of Henle in the setting of AKI [68].	Increased urine OLFM4 is associated with lack of response to furosemide [69]; may identify patients who will be diuretic non-responsive and benefit from RRT.	Limited pediatric data Not clinically available

*Abbreviations* *CPB* cardiopulmonary bypass, *SCr* serum creatinine, *GFR* glomerular filtration rate, *UTI* urinary tract infection, *CKD *chronic kidney disease, *RRT* renal replacement therapy, *CE* Conformite Europeenne, *FDA* Food and Drug Administration

The dynamic changes in biomarkers of AKI with both widespread use (SCr, UOP, fluid balance) and with less availability (novel functional and tubular damage biomarkers) requires more study. This is particularly challenging in pediatrics, as only urine NGAL (ProNephro AKI™) and cystatin C are approved for clinical use (Table 2). Existing diagnostic frameworks for AKI, inclusive of KDIGO and refined KDIGO to include biomarkers, remain static [5, 7]. Meanwhile, as the patient evolves and changes course, often in volatile ways early in hospitalization, adjudication of the change in biomarkers is less well characterized. The change(s), and how the course of the diagnostic biomarkers associates with patient condition and intervention(s), is the subject of theragnostic study [38]. Measuring the kinetic eGFR or change in fluid balance over time indicates the response of the glomerulus and tubular function (respectively) to either interventions and/or the patient course. Similarly, assessment of biomarkers indicative of location, a functional process within the nephron segment (i.e., chloride handling at the Loop of Henle– as is the case with the furosemide stress test), and or even the stage of the AKI disease process itself (initiation, extension, repair) are all possible using markers in a theragnostic manner. Theragnostic markers allow for not only identification of particular subphenotype(s)/endotype(s) of a patient, but also are trackable in response to intervention (example is positron emission tomography (PET) used for metabolic processes in the body or radioisotope labeling in cancer targeting) [38, 70]. Early studies related to nephrotoxin exposure and AKI assessment demonstrates the feasibility of theragnostic biomarker evaluation [71, 72]. Moving beyond the outdated and non-supported (physiologically and pathologically) diagnoses of AKI (i.e., prerenal, intrarenal, postrenal or toxic), integration of biomarkers would usher in a more personalized approach to AKI classification, appropriately representative of the heterogeneity of the syndrome (Figs. 1 and 2). Importantly, given the aforementioned limitations in clinical availability of many of the novel biomarkers examined in a research setting, implementation of these approaches at the bedisde are currently limited to those that employ urine NGAL or cystatin C to refine the current diagnostic framework. More work is needed to bring additional markers capable of providing additional, unique information, to the bedside.

## The forward path – matching phenotype to enriched signature for theragnosis and clinical decision support

Taken together, a vertical step in the management of AKI is possible. Paving the path forward to achieve precision medicine requires an integrated approach – combining existing tools and constructs, some used in new ways, with a more dynamic and individualized approach to the patient (Fig. 1). As mentioned earlier, the importance of risk stratification cannot be understated. The first step is the integration of diagnostic tools to facilitate population enrichment. Through a combination of pragmatic and easy to implement algorithms, the complex and heterogeneous mix of patients with acute critical illness must be separated via AKI risk factors and biomarkers indicative of real-time and ongoing injury propagation to perform prognostic and predictive enrichment. Once the population has been separated by risk of progressive, severe, and persistent AKI (and, most importantly, risks for AKI requiring RRT [AKI-D]), a combination biomarker approach to delineate the unique AKI subphenotype/endotype could be employed (Figs. 1 and 2). This ideal biomarker array would assess degree and extent of injury at the levels of perfusion, filtration, secretion, tubular health, and solute-water handling and then by extension, the risks of injury propagation, to provide a robust AKI subphenotype/endotype, each with their own treatable traits [6]. This biomarker array should absolutely include SCr and UOP, just used in more contemporary ways than the historical precedent of static markers and boundaries designating illness or health. Once in place, the pathophysiologic matching and description of the patient then provides an individualized description which affords two robust opportunities: (1) tracking how the AKI clinical picture changes over time – concordant with subphentoype/endotype (i.e., theragnostics) and (2) acting as clinical decision support to triage patients to different management algorithms, including at the time of discharge from the ICU and/or hospital (Fig. 1). In particular, biomarkers to guide follow-up at the time of discharge from a hospitalization complicated by AKI are sorely needed, especially in pediatrics where progression to CKD is now established to be a common sequela of AKI [73], but a national shortage of pediatric nephrologists makes access to this care challenging. Finally, when assessing the impact of these (and other) management strategies on patient outcomes in future studies, a mandate exists to assess AKI-specific endpoints to ensure we are identifying meaningful, disease-modifying treatments [74]. An example of modernizing the endpoints used is to move away from “RRT Use” as an endpoint for AKI. Just as “mechanical ventilation use” is not an endpoint in acute respiratory distress management algorithms, but used as a process metric, RRT use should be viewed as a balancing metric to the status of kidney health as the patient goes through their ICU course. Apt endpoints include RRT duration, RRT free survival, duration of AKI, and fluid balance related endpoints (i.e., pathologic fluid accumulation affecting other organ function) [74]. A recent description of a single center pathway towards precision management in AKI, demonstrating these steps, provides early justification and proof-of-concept, not just for feasibility, but also for efficacy, patient outcome, and financial value [12]. This example of TAKING FOCUS 2, briefly mentioned above, is further highlighted in detail below as a use case tying together many of the concepts outlined in this review.

### An existing use case of precision medicine in pediatric AKI- TAKING FOCUS 2

As noted above, the TAKING FOCUS 2 study was a single center study that operationalized sequential prognostic and predictive enrichment to risk stratify critically ill children for severe, persistent AKI shortly after PICU admission, and subsequently provide non-mandatory clinical decision support (CDS) around fluid management [11, 12]. In this study, the RAI— a validated severe AKI risk prediction tool— served as the initial strategy for prognostic enrichment. All patients admitted to the PICU were screened 12 h after admission using the RAI, which had been embedded into the electronic health record (EHR) and thus was automatically calculated for each patient. This RAI score resulted in the EHR in real-time for bedside clinicians, facilitating a second level of prognostic enrichment when elevated scores (i.e., those ≥ 8) automatically triggered further screening with urine NGAL measurement. Ultimately, the risk profile and the associated CDS for these RAI + patients was further delineated by their uNGAL concentration: those with values < 150 ng/ml were considered low risk with standard care suggested, while those with values ≥ 150 ng/ml were considered higher risk, with subsequent guidance to consider a furosemide stress test for further risk stratification, along with appropriate fluid restriction and earlier consideration of RRT in patients with a worsening trajectory. In this study, furosemide stress test response also served as a predictive enrichment tool, as those with poor response identified a subset of patients with ongoing tubular dysfunction likely to benefit from earlier initiation of RRT for solute and fluid control to mitigate AKI. Through employment of these interventions, the authors were able to demonstrate improved outcomes for children receiving CRRT at their center over a 2-year period following implementation, including shorter ICU lengths of stay, increased survival, and reduced health care costs [12]. This multi-modal, EHR-embedded, real-time precision medicine approach to AKI mitigation and management is successful clinical example to be built upon in future work.

## Conclusions

In summary, AKI is a complex, heterogeneous syndrome that continues to be plagued by a lack of disease-modifying treatments and reliance on supportive care, in large part due to the static and imprecise nature of the current diagnostic criteria. There are use cases both within pediatric AKI and other heterogeneous syndromes that highlight the ability of dynamic, iterative, precision approaches to risk stratification, diagnosis, and management (i.e., theragnosis) to improve quality of care and patient outcomes. While there remain several important gaps with regards to how and when to use AKI biomarkers, much of what we do know has failed to translate to practice, equating to missed opportunities to leverage the tools we have toward this end of precision AKI management. To improve the care of critically ill children with, at risk for, and following AKI, it is incumbent upon clinicians and researchers alike to move toward a more dynamic, multi-modal, and precise diagnostic framework that also serves to inform treatment.

## Data Availability

Not applicable.
